# Supplementary material for: The use of methylprednisolone in COVID-19 patients: A propensity score matched retrospective cohort study
Source: PLoS One. 2020 Dec 31;15(12):e0244128. doi: 10.1371/journal.pone.0244128 (PMC7775059; doi:10.1371/journal.pone.0244128)
Supplement: S1 Table — (DOCX) [file pone.0244128.s001.docx]

S1 Table. The base line characteristics of the critically ill and non-critically ill COVID-19 patients.

|  | Non-critically ill | | |  | Critically ill | | | |
| --- | --- | --- | --- | --- | --- | --- | --- | --- |
|  | Non-methylprednisolone group | Methylprednisolone group | p-value |  | Non-methylprednisolone group | Methylprednisolone group | p-value | |
|  | (N=216) | (N=60) |  |  | (N=9) | (N=58) |  | |
| Baseline characteristics | | | | | | | |  |
| Sex |  |  |  |  |  |  |  | |
| Female | 100 (46.3%) | 32 (53.3%) | 0.33 |  | 5 (56%) | 20 (34%) | 0.22 | |
| Male | 116 (53.7%) | 28 (46.7%) |  |  | 4 (44%) | 38 (66%) |  | |
| Age, mean (SD) | 50.15 (17.11) | 52.25 (15.09) | 0.39 |  | 68.67 (17.50) | 61.40 (14.44) | 0.18 | |
| Presence of Comorbidities |  |  |  |  |  |  |  | |
| History of tobacco smoking | 35 (16.2%) | 8 (13.3%) | 0.59 |  | 1 (11%) | 14 (25%) | 0.37 | |
| History of alcohol consumption | 19 (8.8%) | 2 (3.3%) | 0.16 |  | 0 (0%) | 3 (5%) | 0.49 | |
| Hypertension | 37 (17.1%) | 13 (21.7%) | 0.42 |  | 5 (56%) | 26 (45%) | 0.55 | |
| Diabetes | 16 (7.4%) | 5 (8.3%) | 0.81 |  | 4 (44%) | 17 (29%) | 0.36 | |
| Coronary heart disease | 6 (2.8%) | 0 (0.0%) | 0.19 |  | 3 (33%) | 11 (19%) | 0.32 | |
| Cerebral cardiovascular disease | 4 (1.9%) | 0 (0.0%) | 0.30 |  | 2 (22%) | 7 (12%) | 0.42 | |
| COPD | 4 (1.9%) | 0 (0.0%) | 0.29 |  | 0 (0%) | 5 (9%) | 0.36 | |
| Malignancy | 3 (1.4%) | 1 (1.7%) | 0.87 |  | 1 (11%) | 4 (7%) | 0.65 | |
| Hepatitis B | 10 (6.9%) | 2 (4.7%) | 0.60 |  | 0 (0%) | 2 (6%) | 0.54 | |
| Hisotry of being in Wuhan | 75 (37.5%) | 30 (53.6%) | 0.031 |  | 0 (0%) | 23 (42%) | 0.015 | |
| History of ever contracting with patients infected with COVID-19 | 53 (46.5%) | 7 (22.6%) | 0.017 |  | 3 (75%) | 10 (37%) | 0.15 | |
| Symptoms and signs | | | | | | | |  |
| Fever | 151 (69.9%) | 53 (88.3%) | 0.004 |  | 8 (89%) | 54 (93%) | 0.65 | |
| Fever as initial symptoms | 131 (60.9%) | 51 (86.4%) | <0.001 |  | 7 (78%) | 43 (75%) | 0.88 | |
| Maximum body temperature (℃) |  |  | <0.001 |  |  |  |  | |
| - <37.3 | 70 (32.4%) | 7 (11.7%) |  |  | 1 (11%) | 3 (5%) | 0.073 | |
| - 37.3-38.0 | 69 (31.9%) | 16 (26.7%) |  |  | 5 (56%) | 9 (16%) |  | |
| - 38.1-39.0 | 73 (33.8%) | 30 (50.0%) |  |  | 3 (33%) | 31 (53%) |  | |
| - >39.0 | 4 (1.9%) | 6 (10.0%) |  |  | 0 (0%) | 11 (19%) |  | |
| - Missing values | 0 (0.0%) | 1 (1.7%) |  |  | 0 (0%) | 4 (7%) |  | |
| Dry cough | 149 (69.0%) | 43 (75.4%) | 0.34 |  | 6 (67%) | 52 (90%) | 0.060 | |
| Expectoration (coughing phlegm) | 79 (36.6%) | 27 (45.0%) | 0.24 |  | 6 (67%) | 42 (72%) | 0.72 | |
| Shortness of breath | 5 (2.3%) | 6 (10.0%) | 0.007 |  | 3 (33%) | 30 (52%) | 0.30 | |
| Headache | 7 (3.2%) | 2 (3.3%) | 0.97 |  | 0 (0%) | 5 (9%) | 0.36 | |
| Fatugue | 57 (26.4%) | 30 (50.0%) | <0.001 |  | 2 (22%) | 31 (53%) | 0.081 | |
| Diarrhea | 9 (4.2%) | 3 (5.0%) | 0.78 |  | 1 (11%) | 0 (0%) | 0.011 | |
| Time from illness onset to first hospital admission (days), median (IQR) | 3 (2, 5) | 3 (2, 5) | 0.73 |  | 4 (2, 4) | 5 (3, 7) | 0.16 | |
| Severity assessment at admission |  |  |  |  |  |  |  | |
| Mild | 6 (2.8%) | 0 (0.8%) | 0.19 |  | 7 (78%) | 26 (45%) | 0.066 | |
| Stable | 210 (97.2%) | 60 (100.0%) |  |  | 2 (22%) | 32 (55%) |  | |
