# Supplementary material for: The use of methylprednisolone in COVID-19 patients: A propensity score matched retrospective cohort study
Source: PLoS One. 2020 Dec 31;15(12):e0244128. doi: 10.1371/journal.pone.0244128 (PMC7775059; doi:10.1371/journal.pone.0244128)
Supplement: S2 Table — (DOCX) [file pone.0244128.s002.docx]

S2 Table. Treatment and outcomes of critically ill and non-critically ill patients with COVID-19 receiving methylprednisolone and non-methylprednisolone therapy.

|  | Non-critically ill | | | | | |  | Critically ill | | | | | | |  |  |
| --- | --- | --- | --- | --- | --- | --- | --- | --- | --- | --- | --- | --- | --- | --- | --- | --- |
|  | Non-methylprednisolone group | | Methylprednisolone group | | p-value | |  | Non-methylprednisolone group | | | Methylprednisolone group | | p-value | |  |  |
|  | (N=216) | | (N=60) | |  | |  | (N=9) | | | (N=58) | |  | |  |  |
| Antiviral Treatment | 216 (100.0%) | | 60 (100.0%) | | / | |  | 9 (100%) | | | 57 (98%) | | 0.69 | |  |  |
| Interferon | 148 (68.5%) | | 44 (73.3%) | | 0.47 | |  | 5 (56%) | | | 19 (33%) | | 0.20 | |  |  |
| Oseltamivir | 158 (73.1%) | | 52 (86.7%) | | 0.030 | |  | 7 (78%) | | | 40 (69%) | | 0.59 | |  |  |
| Arbidol | 105 (48.6%) | | 22 (36.7%) | | 0.10 | |  | 6 (67%) | | | 33 (57%) | | 0.58 | |  |  |
| Lopinavir and ritonavir | 134 (62.0%) | | 55 (91.7%) | | <0.001 | |  | 6 (67%) | | | 41 (71%) | | 0.81 | |  |  |
| Ribavirin | 29 (13.4%) | | 4 (6.7%) | | 0.15 | |  | 1 (11%) | | | 7 (12%) | | 0.93 | |  |  |
| Ganciclovir | 5 (2.3%) | | 1 (1.7%) | | 0.76 | |  | 0 (0%) | | | 4 (7%) | | 0.42 | |  |  |
| Chinese patent medicine |  | |  | |  | |  |  | | |  | |  | |  |  |
| Xuebiqing | 98 (45.4%) | | 33 (55.0%) | | 0.19 | |  | 8 (89%) | | | 45 (78%) | | 0.44 | |  |  |
| Tanreqing | 45 (20.9%) | | 19 (31.7%) | | 0.082 | |  | 3 (33%) | | | 16 (28%) | | 0.72 | |  |  |
| Reduning | 74 (34.4%) | | 14 (23.3%) | | 0.10 | |  | 2 (22%) | | | 18 (31%) | | 0.59 | |  |  |
| Immune enhancer |  | |  | |  | |  |  | | |  | |  | |  |  |
| Thymalfasin | 25 (11.6%) | | 7 (11.7%) | | 0.98 | |  | 4 (44%) | | | 29 (50%) | | 0.76 | |  |  |
| Gamma-immunoglobulin | 10 (4.6%) | | 8 (13.3%) | | 0.016 | |  | 1 (11%) | | | 46 (79%) | | <0.001 | |  |  |
| Ambroxol | 20 (9.3%) | | 7 (11.7%) | | 0.58 | |  | 3 (33%) | | | 26 (45%) | | 0.52 | |  |  |
| Antibacterial treatment | 197 (91.2%) | | 56 (93.3%) | | 0.60 | |  | 9 (100%) | | | 57 (98%) | | 0.69 | |  |  |
| Antifungal treatment | 3 (1.4%) | | 0 (0%) | | 0.36 | |  | 0 (0%) | | | 17 (29%) | | 0.060 | |  |  |
| Duration of methylprednisolone therapy (Days), median (IQR) | 0 (0, 0) | | 4 (3, 6) | | <0.001 | |  | 0 (0, 0) | | | 7 (4, 13) | | <0.001 | |  |  |
| Drug amount of methylprednisolone used, mean (SD) | 0.00 (0.00) | | 223.16 (172.07) | | <0.001 | |  | 0.00 (0.00) | | | 529.11 (356.27) | | <0.001 | |  |  |
| outcomes | | | | | | | | | | | | | | | | |
| Outcomes | |  | |  | |  | | |  |  | |  | |  | |  |
| Discharge | | 216 (100.0%) | | 60 (100.0%) | | / | | |  | 8 (89%) | | 43 (74%) | | 0.37 | |  |
| Death | | 0 (0%) | | 0 (0%) | |  | | |  | 1 (11%) | | 15 (26%) | |  | |  |
| Time taken for Nasopharyngeal swab tests restults to turn negative(days), median (IQR) | | 11 (9, 15) | | 13 (9.5, 18) | | 0.099 | | |  | 12 (10, 17) | | 14 (10, 21.5) | | 0.58 | |  |
| Oxygen support | |  | |  | |  | | |  |  | |  | |  | |  |
| Nasal catheter | | 152 (70.4%) | | 51 (85.0%) | | 0.066 | | |  | 7 (78%) | | 13 (22%) | | 0.009 | |  |
| High-flow oxygen or non-invasive | | 3 (1.4%) | | 0 (0.0%) | |  | | |  | 2 (22%) | | 32 (55%) | |  | |  |
| Invasive | | 0 (0.0%) | | 0 (0.0%) | |  | | |  | 0 (0%) | | 12 (21%) | |  | |  |
| No need | | 61 (28.2%) | | 9 (15.0%) | |  | | |  | 0 (0%) | | 1 (2%) | |  | |  |
| Oxygen saturation ≤93% | | 9 (4.2%) | | 4 (6.7%) | | 0.42 | | |  | 3 (33%) | | 31 (53%) | | 0.26 | |  |
| Acute kidney injury | | 1 (0.5%) | | 1 (1.7%) | | 0.33 | | |  | 2 (22%) | | 6 (10%) | | 0.31 | |  |
| Acute respiratory distress syndrome | | 2 (0.9%) | | 2 (3.3%) | | 0.17 | | |  | 1 (11%) | | 19 (33%) | | 0.19 | |  |
| Use of reduced glutathione | | 29 (13.4%) | | 16 (26.7%) | | 0.014 | | |  | 4 (44%) | | 33 (57%) | | 0.48 | |  |
| Liver protection therapy | | 28 (13.1%) | | 14 (23.3%) | | 0.051 | | |  | 5 (56%) | | 33 (57%) | | 0.94 | |  |
